# Partial mitochondrial genome of the enigmatic Bermuda fireworm Odontosyllis enopla Verrill, 1900 (Annelida, Syllidae, Eusyllinae) and its phylogenetic implications

**DOI:** 10.3897/zookeys.1270.177446

**Published:** 2026-02-19

**Authors:** Lynette D. Wyant, Brendan A. Cruz, Aydanni D. Gonzalez, Joshua M. Kovalcik, Maria A. Carolus, Lauren C. Hutto, Hope Chutjian, Jude C. Roman, Anneau Cappelmann, John J. Ankney, Aidan Popp, James B. Wood, D. Tye Pettay, Mercer R. Brugler

**Affiliations:** 1 Department of Natural Sciences, University of South Carolina Beaufort, 1100 Boundary St, Beaufort, SC 29902, USA National Museum of Natural History, Smithsonian Institution Washington United States of America; 2 Hawai‘i Institute of Marine Biology, University of Hawai‘i at Mānoa, Kāne‘ohe, HI 96744, USA American Museum of Natural History New York United States of America; 3 Division of Invertebrate Zoology, American Museum of Natural History, Central Park West at 79th Street, New York, NY 10024, USA University of South Carolina Beaufort Beaufort United States of America; 4 Department of Invertebrate Zoology, National Museum of Natural History, Smithsonian Institution, 10th St. & Constitution Ave. NW, Washington, DC 20560, USA University of Hawai‘i at Mānoa Kāne‘ohe United States of America

**Keywords:** Bioluminescent, gene overlap, intraspecific variation, mitogenome, molecular phylogeny, origin of replication, polychaete

## Abstract

The Bermuda fireworm, *Odontosyllis enopla* Verrill, 1900, is a marine polychaete that displays a unique bioluminescent mating ritual. Despite the first sighting of *O. enopla* more than 534 years ago, molecular data have been limited. Several syllid mitogenomes are currently available; however, there are only three published genes for *O. enopla*: two partial mitochondrial genes (16S [508 bp] and *cox1* [653 bp]; 1,161 bp total) and one partial nuclear gene (18S [1,339 bp]). This study bioinformatically mined previously published transcriptomes of *O. enopla* for mitochondrial reads and subsequently assembled and annotated a partial mitochondrial genome (10,172 bp). The partial mitogenome includes nine (of 13) protein-coding genes, two ribosomal RNAs, and seven (of 22) complete tRNAs. We place the Bermuda fireworm in phylogenetic context using all available syllid mitogenomes, analyze intraspecific variation among three female *O. enopla* partial mitogenomes, and propose a putative location for the mitochondrial origin of replication using a DNA Walker analysis.

## Introduction

**Figure 1.**
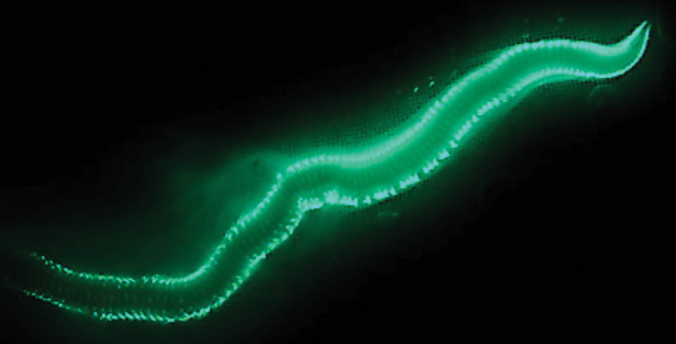
*Odontosyllis enopla* (image credit: coauthor James B. Wood). Photo taken near the shore at the north point of Ferry Island, St. George’s Parrish, Bermuda.

*Odontosyllis enopla* Verrill, 1900, more commonly known as the Bermuda fireworm, is a remarkable annelid belonging to the family Syllidae (Fig. 1). These tube-dwelling worms, found on sandy coral substrates in benthic habitats (Fischer and Fischer 1995), display an incredible bioluminescent mating ritual that was first seen and recorded by Christopher Columbus in 1492 (Brugler et al. 2018) and first described by Galloway and Welch (1911).

The Bermuda fireworm is a unique annelid due to its distinct mating behaviors and physiology. In preparation for breeding, Bermuda fireworms of both sexes undergo morphological changes, including enlargement and pigmentation of their four eyes to enhance visual sensitivity. This is particularly pronounced in males for detecting the females’ bioluminescence (Wolken and Florida 1984; Franke 1999). This sets the stage for a highly synchronized mating swarm, where selective pressures from predation favor precise timing to minimize vulnerability of isolated or early individuals (Huntsman 1948). Females initiate the ritual by rising to the surface and swimming in slow circles while emitting a continuous bluish-green glow from secreted luminous mucus and releasing their gametes. Triggered by this display, males swim towards the glowing females, producing quick, consecutive bioluminescent flashes while releasing their own gametes into the surrounding water (Fischer and Fischer 1995; Verdes et al. 2022). The bioluminescence peaks in the green portion of the visible spectrum, with wavelengths between 504–507 nm. Their visual system is most sensitive to the aforementioned wavelength, as shown by electroretinogram (ERG) recordings in response to light at multiple wavelengths (Wilkens and Wolken 1981). The similarities between the light detected and the light emitted suggest that the Bermuda fireworms are visually tuned to detect mating signals from bioluminescence.

The Bermuda fireworm synchronizes its mating ritual with the lunar cycle, with swarming episodes coinciding with the first day after full moons during the summer and early autumn months (Brugler et al. 2018). Utilizing specialized chaetae, this benthic organism will swim to the surface 57 ± 1 minutes after the astronomical sunset to begin its mating ritual (Fischer and Fischer 1995). This mating swarm has been observed up to five nights following the full moon, after which both male and female Bermuda fireworms return to their benthic habitats (Fischer and Fischer 1995). The fertilized zygotes undergo cell division and, after 14 hours, become trochophores that can swim freely through the water column (Fischer and Fischer 1995).

Evidence suggests that the common ancestor of all bioluminescent syllid species was not bioluminescent itself; in fact, bioluminescence in *Odontosyllis* Claparède, 1863 evolved independently twice (Verdes et al. 2022). While the bioluminescence of this species is of great interest to researchers, the *reversible* epitoky metamorphosis in both males and females is of even greater importance. The Bermuda fireworm undergoes different physiological changes to prepare for mating, including enlargement of the eyes in males, the growth of chaetae used for rapid swimming to reach the surface, and tissues protruding from the body of the females holding the oocytes (Fischer and Fischer 1995; Brugler et al. 2018). After the mating swarm, the swimming chaetae are shed, although there appears to be no specific timeline for this reversal. Some Bermuda fireworms shed the chaetae in as little as five days after swarming, while others had at least some remaining chaetae after 35 days. Additional research needs to be conducted regarding the reversal of the size of the male eyes. Research thus far indicates that there was no reduction in male eye size two months after swarming and therefore may simply reflect sexual maturity (Fischer and Fischer 1995).

To date, only three genes have been published for the Bermuda fireworm, totaling 2,500 base pairs (bp). Of the three genes published, two are mitochondrial genes: the large subunit ribosomal RNA (16S) and cytochrome *c* oxidase subunit I (*cox1*). The third gene is the nuclear small subunit ribosomal RNA (18S). The majority of available mapped mitochondrial genomes for annelids are from the clades Errantia and Sedentaria. The family Syllidae, of which the Bermuda fireworm is a member, is known to have a highly variable mitochondrial genome in terms of gene order (Aguado et al. 2016; Schwarze et al. 2026). This manuscript presents the partial mitochondrial genome for the Bermuda fireworm and places it in phylogenetic context amongst its relatives. To our knowledge, the only study that has included *Odontosyllis enopla* in a phylogenetic context was a three-gene phylogeny by Verdes et al. (2022) where the authors listed *O. enopla* under the species ID ‘*Odontosyllis* sp. 9’ and specimen code ‘OenoTR.’ We also analyze intraspecific variation among three female *O. enopla* partial mitogenomes and propose a putative location for the mitochondrial origin of replication, which, to date, has not been definitively identified in the Bermuda fireworm or its closest relatives.

## Material and methods

### Background on transcriptome acquisition

Per Brugler et al. (2018), total RNA was isolated from the whole body of three female *Odontosyllis enopla* worms using a modified RNeasy Tissue Kit (Qiagen) protocol (voucher material not available as worms were completely macerated). Isolates were prepared using the TruSeq Stranded mRNA Library Prep Kit (Illumina, San Diego, CA) with a 350 bp insert size and run at the NY Genome Center on an Illumina HiSeq 2500 (2 × 125 bp), allocating 1/8 of a lane for each isolate. The run generated 37,063,191 (Individual #1), 39,513,743 (Individual #2), and 34,329,885 (Individual #3) raw reads. After trimming adaptors and low-quality regions, assembly with Trinity yielded 176,598 (Individual #1), 207,006 (Individual #2) and 283,041 (Individual #3) contigs (including splice variants). These represented 44,426 (Individual #1), 49,458 (Individual #2) and 61,002 (Individual #3) open reading frames (>100 amino acids) predicted by Transdecoder and included >99.0% of the 2,748 core KOGs.

### Bioinformatics

Mitochondrial reads were bioinformatically extracted from the transcriptomes of three female *Odontosyllis enopla* worms using MitoFinder v. 1.4 (Allio et al. 2020). MitoFinder employed MEGAHIT v. 3.0 (Li et al. 2015) for mitogenome assembly and tRNAscan-SE (Chan and Lowe 2019) for tRNA annotation. The following command was used to run MitoFinder on an iMac: ./mitofinder --megahit --override --new-genes -j [file name] -1 [left_reads.fastq.gz] -2 [right_reads.fastq.gz] -r [genbank_reference.gb] -o [genetic_code] -p [threads] -m [memory] -t trnascan. *Eusyllis blomstrandi* Malmgren, 1867 (GenBank accession no. KX752423; 14,712 bp in length) was used as the reference, and Translation Table 1 (Invertebrate Mitochondrial Code) was used as the genetic code. We would have preferred to use *Odontosyllis undecimdonta* Imajima & Hartman, 1964 as the reference, but only two mitochondrial genes were available on GenBank in 2025 (16S and *cox1*). Newly assembled partial mitogenomes were annotated using the MITOS Web Server (Bernt et al. 2013). Of the three *Odontosyllis enopla* worms, individual #3 (specimen ID: oe3) yielded the longest single mitochondrial contig at 10,172 bp, and thus this partial mitogenome is described herein. We used MEGA X (Kumar et al. 2018; Stecher et al. 2020) to obtain intraspecific genetic distance estimates (*p*-distances) among the three partial *O. enopla* mitogenomes.

**Table 1.** Gene order and length of *Odontosyllis enopla* mitochondrial protein-coding genes, ribosomal RNAs, transfer RNAs, and intergenic regions (IGRs).

Gene	Length (bp)
12S	607
tRNA-Val	62
IGR	221
16S	730
IGR	31
tRNA-Leu	62
IGR	123
tRNA-Ala	62
IGR	442
*NAD1*	924
tRNA-Ile	61
IGR	3
tRNA-Lys	63
IGR	2
*NAD3*	353
IGR	60
*NAD2*	921
IGR	81
*COX1*	1533
IGR	45
*COX2*	684
IGR	64
*ATP8*	162
tRNA-Tyr	62
COX3**	780
tRNA-Gln**	61
IGR	2
NAD6*	504
COB*	1131
IGR	28

*NAD6 overlaps with COB. **COX3 overlaps with tRNA-Gln.

### Phylogenetic analysis

The partial mitogenome of *Odontosyllis enopla* (GenBank accession no. PP998669) was combined with mitogenomes presented in Aguado et al. (2015a; *Ramisyllis multicaudata* Glasby, Schroeder & Aguado, 2012 and *Trypanobia cryptica* Aguado, Murray & Hutchings, 2015c), Aguado et al. (2016; *Eusyllis blomstrandi*, *Myrianida brachycephala* (Marenzeller, 1874), *Streptosyllis* sp. Webster & Benedict, 1884, *Typosyllis antoni* Aguado, Helm, Weidhase & Bleidorn, 2015b, and *Typosyllis* sp. (Langerhans, 1879)), Aguado et al. (2022; *Ramisyllis kingghidorahi* Aguado, Ponz-Segrelles, Glasby, Ribeiro, Jimi & Miura, 2022 [in Aguado et al. 2022]), Cejp et al. (2023; *Clavisyllis tenjini* Cejp, Jimi & Aguado, 2023), and Chae et al. (2023; *Syllis* sp. Lamarck, 1818) for a total of 11 mitogenomes. Each of the nine protein-coding genes (*cob*, *atp8*, *cox1-3*, *nad1-3*, *nad6*) and two ribosomal RNAs (12S and 16S) from all 11 mitogenomes were placed in individual AliView v. 1.23 (Larsson 2014) files and individually aligned using MAFFT LINS-i v7 (Katoh et al. 2019). GBlocks v. 0.91b was applied to each individual gene region to remove poorly aligned positions and divergent regions. Each individual gene region was subsequently concatenated into a single file using Seqotron v. 1.0.1 (Fourment and Holmes 2016), treating the mitogenome as a single locus. GBlocks reduced the length of the multiple sequence alignment to 7,097 bp (alignment available upon request to the corresponding author MRB).

The Akaike Information Criterion (AIC) within jModelTest v. 2.1.10 (Guindon and Gascuel 2003; Darriba et al. 2012) selected the GTR+I+G model of sequence evolution (p-inv: 0.2800; gamma: 1.0260). A maximum-likelihood-based phylogenetic tree was built using the command-line version of PhyML v. 3.1 (Guindon et al. 2010). PhyML parameters included a tree topology search consisting of the best of NNIs and SPRs, a BioNJ starting tree, and 1,000 bootstrap replicates. The resulting phylogenetic tree was visualized using FigTree v. 1.4.4 (by Andrew Rambaut; https://github.com/rambaut/figtree/releases). The tree was rooted with *Streptosyllis* sp. (KX752422) based on a phylogenetic analysis conducted by Aguado et al. (2016) using full mitogenomes (but see DeSalle et al. 2023).

### Origin of replication

**Figure 2.**
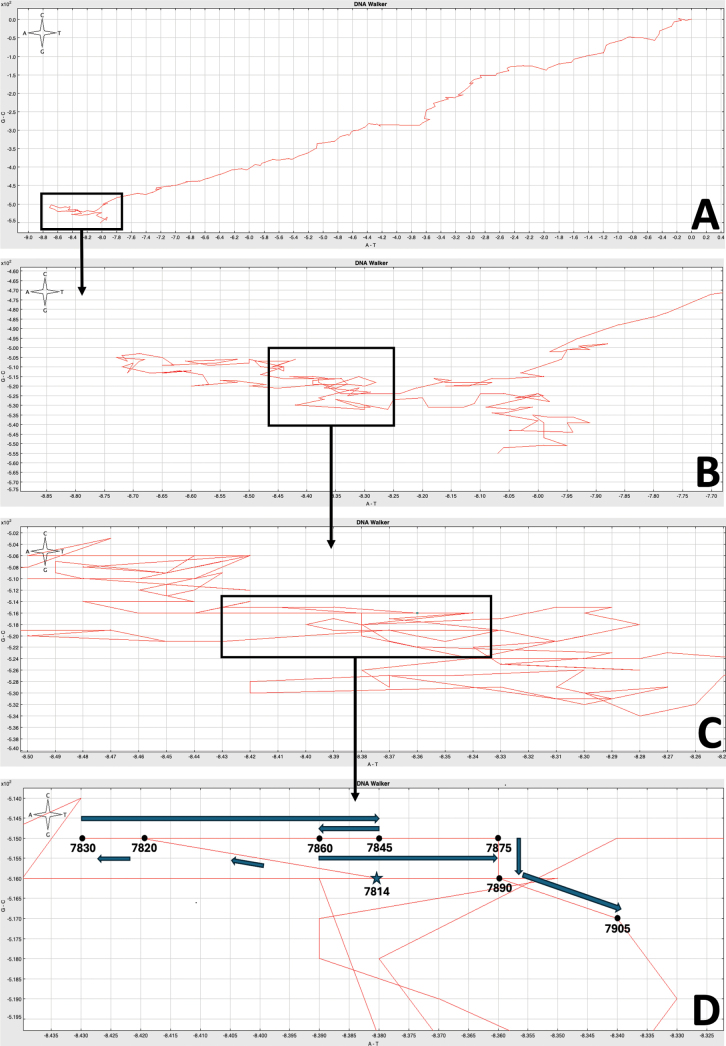
A DNA Walk of the partial mitochondrial genome of *Odontosyllis enopla*. Abrupt changes in base composition bias (switchbacks) are characteristic of the origins of replication. **A**. Full mitogenome walk (window size: 75); **B**. Zoomed-in image of switchback (window size: 20); **C**. Zoomed-in image of switchback (window size: 10); **D** Zoomed-in image of switchback with nucleotide positions and cardinal direction changes indicated (window size: 15).

**Figure 3.**
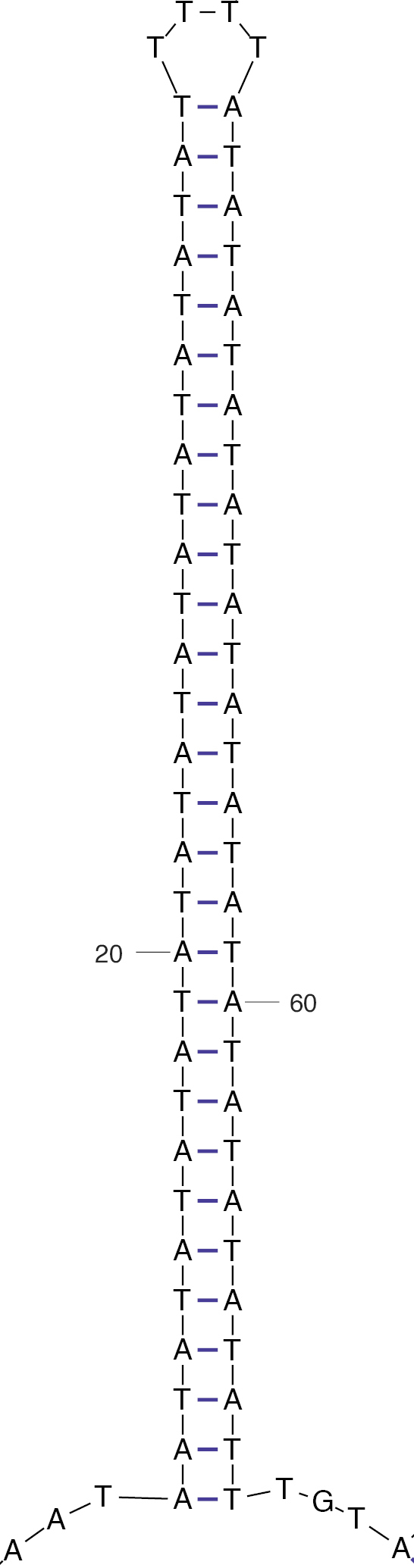
The most thermodynamically stable stem-loop structure (dG = −22.02) as output by the UNAFold web server. Note the long AT-rich stem containing a characteristic T-rich loop, which is a common feature within the origin of replication.

The DNA Skew Graphing tool (GraphDNA; Thomas et al. 2007), available online via the Viral Bioinformatics Research Centre (https://4virology.net/), was used to search representative mitochondrial genomes for abrupt changes in base composition bias characteristic of the origin of replication. In particular, we used the ‘DNA Walker’ graphing option (per Lobry 1996; Fig. 2). After locating a putative origin of replication, we used the default parameters in the DNA Folding Form on the UNAFold web server (Zuker 2003) to identify a stable stem-loop configuration containing a characteristic T-rich loop (Fig. 3), a common feature of origins of replication.

## Results

**Figure 4.**
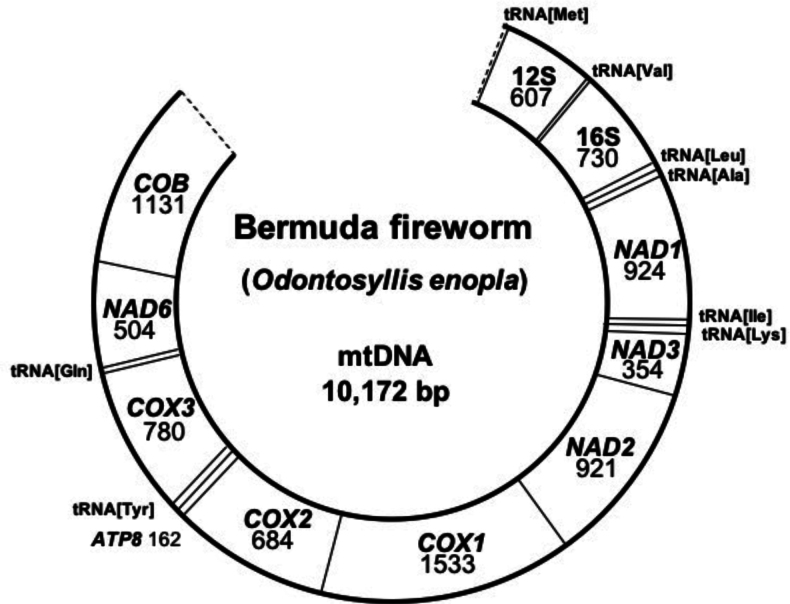
Partial mitochondrial genome map of *Odontosyllis enopla*. We were unable to bioinformatically recover *nad4L*, *atp6*, and *nad4*-*5*.

**Figure 5.**
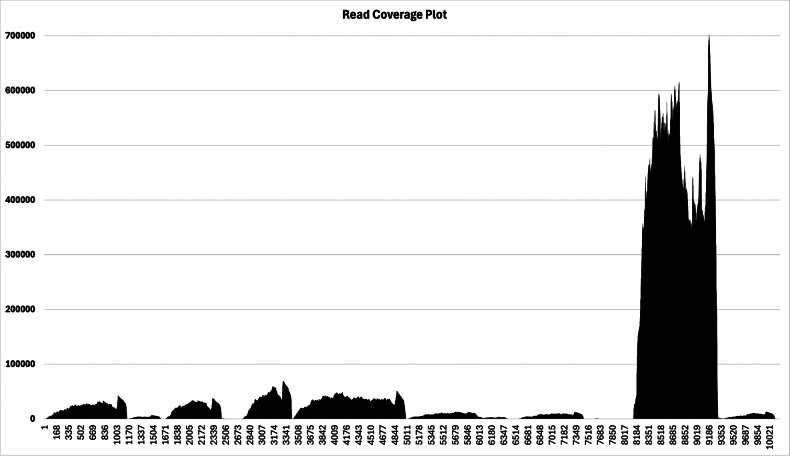
A read coverage plot showing the number of individual reads (*y*-axis) that mapped to the different parts of the assembled mitogenome (*x*-axis; 10,172 bp in length).

**Figure 6.**
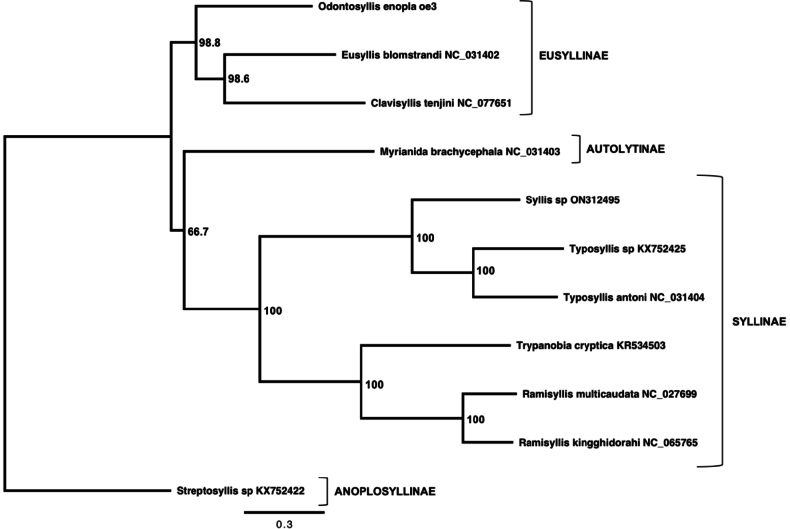
A maximum-likelihood-based phylogenetic tree based on nine protein-coding genes (*cob*, *atp8*, *cox1*-*3*, *nad1*-*3*, *nad6*) and two ribosomal RNAs (12S, 16S). Node support is based on 1,000 bootstrap replicates. The tree was rooted with *Streptosyllis* sp. based on a phylogenetic analysis by Aguado et al. (2016) using complete mitogenomes. The following sequences were used: *Odontosyllis enopla*PP998669 (this study), *Ramisyllis multicaudata*NC_027699 and *Trypanobia cryptica*KR534503 (Aguado et al. 2015a), *Eusyllis blomstrandi*NC_031402, *Myrianida brachycephala*NC_031403, *Streptosyllis* sp. KX752422, *Typosyllis antoni*NC_031404, and *Typosyllis* sp. KX752425 (Aguado et al. 2016), *Ramisyllis kingghidorahi*NC_065765 (Aguado et al. 2022), *Clavisyllis tenjini*NC_077651 (Cejp et al. 2023), and *Syllis* sp. ON312495 (Chae et al. 2023).

The partial mitogenome of the Bermuda fireworm, *Odontosyllis enopla*, is 10,172 bp in length and contains nine of the 13 protein-coding genes (*cob*, *atp8*, *cox1-3*, *nad1-3*, *nad6*), two ribosomal RNAs (12S, 16S) and eight transfer RNAs (Met, Val, Leu, Ala, Ile, Lys, Tyr, Gln) (Fig. 4). We were unable to bioinformatically recover *nad4L*, *atp6*, and *nad4-5*. The partial mitogenome is available under GenBank accession no. PP998669. Gene order for the partial mitogenome of *Odontosyllis enopla* is as follows: tRNA[Met]-12S-tRNA[Val]-16S-tRNA[Leu]-tRNA[Ala]-*nad1*-tRNA[Ile]-tRNA[Lys]-*nad3*-*nad2*-*cox1*-*cox2*-*atp8*-tRNA[Tyr]-*cox3*-tRNA[Gln]-*nad6*-*cob* (Table 1). Of the genes recovered, the gene order of *O. enopla* matches that of *Eusyllis blomstrandi* (GenBank accession no. NC_031402; the *E. blomstrandi* mitogenome is 14,712 bp in length). We are missing the following gene segment: *atp6*-*nad5*-*nad4L*-*nad4*. MitoFinder found no evidence of circularization of the 10,172 bp fragment. A read coverage plot is presented in Fig. 5. The 16S ribosomal RNA (position 8182–9304) was the most transcriptionally active gene (upwards of 700K reads per position), followed by cytochrome *c* oxidase subunit II (*cox2*; position 2749–3432; maximum of ~70K reads per position) and cytochrome *c* oxidase subunit I (*cox1*; position 3458–5008; maximum of ~50K reads per position). Cells typically manufacture significant amounts of ribosomal RNA (16S and 12S) and can terminate transcription after these genes are successfully copied. Interestingly, 12S ribosomal RNA (position 9386–9984) was one of the least transcriptionally active genes. Similar to other invertebrates, the *O. enopla* mitogenome is AT-rich (A: 3,880, T: 3,075, G: 1,886, C: 1,331). We obtained 6,120 bp of comparable sequence data from the three *O. enopla* mitogenomes, yielding eight variable sites. Individual #1 (specimen ID: oe1) had four unique substitutions, individual #2 (specimen ID: oe2) had three unique substitutions, and individual #3 (specimen ID: oe3) had one unique substitution (Table 2). A maximum-likelihood-based phylogenetic tree based on nine protein-coding genes (*cob*, *atp8*, *cox1-3*, *nad1-3*, *nad6*) and two ribosomal RNAs (12S, 16S) showed *Odontosyllis enopla* grouping sister to a clade comprised of *Eusyllis blomstrandi* (NC_031402) and *Clavisyllis tenjini* (NC_077651) with 98.8 node support (Fig. 6).

**Table 2.** Genetic distance estimates (*p*-distances) among the three *Odontosyllis enopla* mitogenomes (based on 6,120 bp of comparable sequence data). A total of eight variable sites were identified. Individual #1 (specimen ID: oe1); Individual #2 (specimen ID: oe2); Individual #3 (specimen ID: oe3).

	oe1	oe2
**oe1**	—	—
**oe2**	0.00114 (= 0.114%)	—
**oe3**	0.000817 (= 0.0817%)	0.000654 (= 0.0654%)

## Discussion and conclusion

Prior to this publication, only two partial mitochondrial sequences for *Odontosyllis enopla* were available in GenBank: the large subunit ribosomal RNA gene (16S; 508 bp) and the cytochrome *c* oxidase subunit 1 gene (*cox1*; 653 bp). Combined, these sequences are 1,161 bp in length. Our newly obtained sequence data (10,172 bp) represent more than 8.76 times the amount of mitochondrial DNA than was previously available.

*Odontosyllis enopla* was included in a three-gene phylogeny by Verdes et al. (2022), where the authors listed *O. enopla* under the species ID “*Odontosyllis* sp. 9” and specimen code “OenoTR”. In that phylogeny, *Odontosyllis* was recovered as paraphyletic. *O. enopla* and congeners (“Clade 2” per Verdes et al. (2022)) are grouped sister to *Nudisyllis* Knox & Cameron, 1970. The clade consisting of “Clade 2” *Odontosyllis* + *Nudisyllis* is grouped sister to “Clade 1” *Odontosyllis* + *Eusyllis* + *Pionosyllis* Malmgren, 1867. Mitochondrial genomes of *Nudisyllis* and *Pionosyllis* were not available at the time of this analysis; however, the complete mitogenome of *Eusyllis blomstrandi* (GenBank accession no. NC_031402) was available and included in our phylogenetic analysis.

The phylogenetic reconstruction placed *Odontosyllis enopla* as a sister (ML bootstrap support: 98.8) to a clade containing *Eusyllis blomstrandi* (NC_031402) and *Clavisyllis tenjini* (NC_077651). These three species are all classified in the subfamily Eusyllinae. Additionally, all three taxa share the same mitochondrial gene order. The topology of our phylogenetic reconstruction was also recovered by Schwarze et al. (2026), who presented a 26-taxon ML tree based on all 13 mitochondrial protein-coding genes, mitochondrial 16S and 12S, and nuclear 18S and 28S. Their phylogeny included two additional members of Autolytinae (*Virchowia christophi* Aguado, Springer, Oguchi, Sato, Jimi & Miura, 2025 [in Springer et al. 2025] and *Myrianida* sp. Milne Edwards, 1845) and nine additional members of Syllinae (*Eurysyllis tuberculata* Ehlers, 1864, *Trypanosyllis* sp. Claparède, 1864, *Parasphaerosyllis ezoensis* Imajima & Hartman, 1964, *Syllis malaquini* Ribeiro, Ponz-Segrelles, Helm, Egger & Aguado, 2020, *Haplosyllis* sp. Langerhans, 1879, *Syllis maganda* Martínez & San Martín, 2020, *Syllis okadai* Fauvel, 1934, *Paraopisthosyllis rufa* Springer, Aguado, Sato, Oguchi, Jimi & Miura, 2025, and *Megasyllis nipponica* (Imajima, 1966)).

We were unable to bioinformatically recover *nad4L*, *atp6*, and *nad4-5*. These four genes are found in tandem (*atp6*-*nad5*-*nad4L*-*nad4*) in the mitogenomes of *Clavisyllis tenjini* (NC_077651), *Eusyllis blomstrandi* (NC_031402), *Myrianida brachycephala* (NC_031403; Autolytinae), and *Streptosyllis* sp. (KX752422; Anoplosyllinae). In *C. tenjini*, *E. blomstrandi*, and *M. brachycephala*, these four genes are located between *cob* and 12S. In *Streptosyllis* sp., these four genes have been translocated between *cox2* and *cox3*. In *Ramisyllis multicaudata* (NC_027699) and *Trypanobia cryptica* (KR534503), both members of Syllinae, *nad5* has been split from the 4-gene segment and moved to a different location (between *cox3* and 16S). In *Typosyllis antoni* (NC_031404) and *Typosyllis* sp. (KX752425), also members of Syllinae, both *nad5* and *atp6* have been split from the 4-gene segment and moved to different locations (*nad5* is between *nad3* and *nad1*, while *atp6* is between 12S and *cox1*). Given the considerable variability in the structure and placement of this four-gene segment across these mitogenomes, these four genes may have been lost during a rearrangement event. That said, MitoFinder found no evidence of circularization. A more plausible explanation is that this four-gene segment (*atp6*-*nad5*-*nad4L*-*nad4*) was not transcriptionally active when the three female *Odontosyllis enopla* worms were collected and preserved. These results also suggest that the four-gene segment may not play a significant role in bioluminescence display, gamete formation, or sexual reproduction more broadly.

According to Patra et al. (2016), most annelid mitochondrial control regions (i.e. the origin of replication or D-Loop) are located between tRNA[Arg] and tRNA[His], but the position does indeed vary in some orders. We searched the nine (of 13) protein-coding genes, two ribosomal RNAs, and seven (of 22) complete tRNAs (totaling 10,172 bp) for evidence of a putative origin of replication. A DNA Walk analysis identified a switchback in cardinal direction in a ~440 bp non-coding region between tRNA[Ala] and *nad1* (Fig. 2). After identifying this putative origin of replication, we used the default parameters in the DNA Folding Form on the UNAFold web server to locate a lengthy stem-loop configuration containing a characteristic T-rich loop (Fig. 3), which is a common feature within the origin of replication. Excluding the tRNAs Leucine and Alanine, the putative control region in *O. enopla* is located between 16S and *nad1*. This placement is notable as Cejp et al. (2022) sequenced the complete mitochondrial genome of four species within the family Chrysopetalidae (Errantia, Phyllodocida, Nereidiformia) and also located the control region between 16S and *nad1* (more specifically, between tRNA[Ile] and tRNA [Leu1] or tRNA [Leu1] and tRNA [Ser2]). Aguado et al. (2016: 93) noted that “In the five mt genomes (*Streptosyllis* sp., *Eusyllis blomstrandi*, *Myrianida brachycephala*, *Typosyllis antoni* and *Typosyllis* sp.), the longest non-coding regions are AT-rich and are suggested to be the putative control regions.” In *E. blomstrandi* and *M. brachycephala*, these regions are located between 12S and 16S (specifically, between tRNA[Val] and 16S). As noted earlier, cells typically manufacture significant amounts of ribosomal RNA (16S and 12S) and can terminate transcription after these genes are successfully copied. Thus, any gene rearrangement that places the origin of replication immediately upstream of ribosomal 16S and 12S genes would have a selective advantage.

We analyzed intraspecific variation among three female *O. enopla* partial mitogenomes and identified eight variable sites across 6,120 bp of comparable sequence data. Individual #1 (specimen ID: oe1) had four unique substitutions, individual #2 (oe2) had three unique substitutions, and individual #3 (oe3) had one unique substitution. Genetic distance estimates (*p*-distances) ranged from 0.114% (comparing oe1 and oe2) to 0.0654% (comparing oe2 and oe3). Within the molecular-based literature on annelids, intraspecific genetic divergences based on mitochondrial DNA are only available for 16S and *cox1* (e.g. Nygren et al. 2010; Aguado et al. 2019; Del Olmo et al. 2024); thus, no comparable data currently exist at the partial or whole mitogenome level. Although not directly comparable, intraspecific genetic distances (uncorrected Kimura two-parameter) within the *Syllis prolifera* Krohn, 1852 species complex ranged from 0.2 ± 0.2 to 0.9 ± 0.2 for 16S and 0.3 ± 0.1 to 1.5 ± 0.2 for *cox1* (Del Olmo et al. 2024). Intraspecific genetic divergences (*p*-distances) within *Amblyosyllis* Grube, 1857 were <1% for 16S (with one exception being 1.9%) and 0–4% for *cox1* (Aguado et al. 2019). According to Kvist S (2016), the median value for intraspecific and interspecific distances within annelid mitochondrial *cox1* is 3.56%, and 20.06%, respectively. We suggest that future studies determine if these thresholds also hold true at the whole mitogenome level.

## Additional information

### Use of AI

No use of AI was reported.

### Author ORCIDs

Lynette D. Wyant https://orcid.org/0009-0001-7700-6940

Brendan A. Cruz https://orcid.org/0009-0008-4422-6489

Aydanni D. Gonzalez https://orcid.org/0009-0007-7049-1019

Joshua M. Kovalcik https://orcid.org/0009-0004-4425-5096

Maria A. Carolus https://orcid.org/0009-0007-6980-4292

Lauren C. Hutto https://orcid.org/0009-0002-7307-6308

Hope Chutjian https://orcid.org/0009-0008-5821-9335

Jude C. Roman https://orcid.org/0009-0002-9297-8008

Anneau Cappelmann https://orcid.org/0009-0007-8700-5726

John J. Ankney https://orcid.org/0009-0002-5983-5523

Aidan Popp https://orcid.org/0009-0009-2354-1399

D. Tye Pettay https://orcid.org/0000-0002-2060-3226

Mercer R. Brugler https://orcid.org/0000-0003-3676-1226

### Data availability

Mitogenomic data are available in GenBank of NCBI (https://www.ncbi.nlm.nih.gov) under accession no. PP998669. The phylogenetic tree can be found on FigShare at the following URL: https://doi.org/10.6084/m9.figshare.30138370. The transcriptomic sequence data that support the findings of this study are openly available in GenBank of NCBI under BioProject accession no. PRJNA448700, SRA accession numbers SRX4382063–SRX4382065, and BioSample accession numbers SAMN08863502, SAMN08863511, and SAMN08863514.
